# Spatial Transcriptomics As Rasterized Image Tensors (STARIT) characterizes cell states with subcellular molecular heterogeneity

**DOI:** 10.64898/2025.12.18.695193

**Published:** 2025-12-22

**Authors:** Dee Velazquez, Caleb Hallinan, Roujin An, Kalen Clifton, Jean Fan

**Affiliations:** 1 Center for Computational Biology, Whiting School of Engineering, Johns Hopkins University, Baltimore, Maryland, USA; 2 Department of Biomedical Engineering, Johns Hopkins University, Baltimore, Maryland, USA

**Keywords:** spatial transcriptomics, rasterization, image analysis, feature extraction, deep learning

## Abstract

**Motivation:**

Imaging-based spatially resolved transcriptomics (imSRT) technologies provide high-throughput molecular-resolution spatial characterization of genes within cells. Conventional analysis methods to identify cell-types and states in imSRT data rely on gene count matrices derived from tallying the number of mRNA molecules detected for each gene per segmented cell, thereby overlooking subcellular heterogeneity that can be useful in defining cell states.

**Results:**

To take advantage of the molecular-resolution information in imSRT data and potentially identify cell-states based on subcellular heterogeneity, we developed STARIT (Spatial Transcriptomics As Rasterized Image Tensors). STARIT converts transcripts within segmented cells in imSRT data into an image-based tensor representation that can be combined with deep learning computer vision models for downstream analysis. Using simulated data, we demonstrate that STARIT distinguishes transcriptionally distinct cell-types and further separates cell states based on subcellular transcript localization, which conventional gene count analysis fails to capture. Likewise, using real imSRT data, we demonstrate how STARIT identifies comparable cell-types to conventional gene count analysis as well as delineate rotational variation. By providing a standardized framework to encode subcellular molecular information in imSRT data, STARIT will enable deeper insights into subcellular heterogeneity and enhance the identification and characterization of cell-types and states that are overlooked by gene count representations.

**Availability and Implementation:**

STARIT is available as a Python package on GitHub at https://github.com/JEFworks-Lab/STARIT.

## 1 Introduction

Recent advances in spatial transcriptomics technologies have enabled spatially resolved gene expression profiling for fixed cells in culture and thin tissue sections. In particular, imaging-based spatially resolved transcriptomics (imSRT) technologies, including MERFISH, seqFISH, Xenium, and others, enable researchers to pinpoint the location of individual mRNA transcripts for targeted genes within cells (Zhuang, 2021). Unlike traditional fluorescence in situ hybridization, where a fluorophore’s color directly encodes the gene identity of a labeled molecule in an image, imSRT methods generally do not assign a detected transcript’s gene identity from a single image alone. Instead, imSRT methods decode a transcript’s gene identity based on multiple rounds of multiplexed imaging. The resulting output is typically represented as a table of decoded transcripts with associated spatial coordinates. To delineate cell boundaries, computational cell segmentation can be applied based on nuclear or membrane stains, total transcript density, or transcript composition (Stringer et al., 2021; Petukhov et al., 2022).

**Figure 1.**
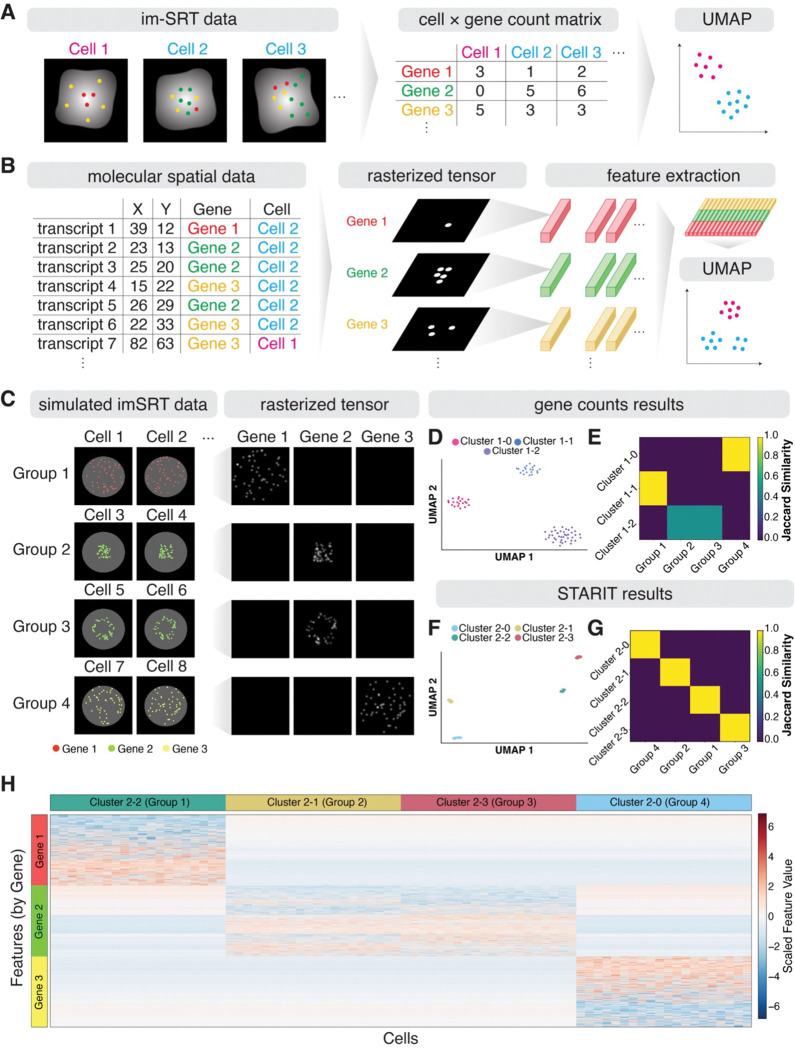
Overview of Spatial Transcriptomics As Rasterized Tensors (STARIT) (A) Schematic of conventional imaging-based spatially resolved transcriptomics (imSRT) data analysis pipeline. Detected transcripts are aggregated into a cell x gene count matrix, which is subsequently used for dimensionality reduction and clustering. Left: Three cells from imSRT data. Middle: Corresponding gene counts matrix. Right: Dimensionality reduction and clustering of imSRT identify distinct cell-types. (B) STARIT (Spatial Transcriptomics As Rasterized Image Tensors) workflow for imSRT data. Left: Molecular representation of imSRT data. Middle: Rasterized image representations for each gene in a cell. Right: Rasterized tensors are then input into a pretrained convolutional neural network (CNN; e.g., ResNet101) for spatial feature extraction. Extracted features are concatenated together to generate a total feature matrix that preserves both molecular and spatial information for downstream clustering and analysis. (C) Simulated imSRT data comprises four groups of cells (Groups 1–4) that exhibit distinct transcriptional and subcellular heterogeneity. Left: Example simulated cells from each group, showing expression of Gene 1 (red), Gene 2 (green), or Gene 3 (yellow). Groups are defined by distinct gene expression (Groups 1 and 4) or distinct subcellular expression patterns of the same gene (nuclear vs. perinuclear; Groups 2 and 3). Right: Rasterized tensor representations of each gene for one simulated cell per group. (D) UMAP visualization based on gene count analysis, with embedding colored by Louvain cluster assignment. (E) Jaccard similarity heatmap comparing ground-truth and gene count-based Louvain clusters. (F) UMAP visualization based on STARIT output, with embedding colored by Louvain cluster assignment. (G) Jaccard similarity heatmap between ground-truth and STARIT-based Louvain clusters. (H) Heatmap of scaled STARIT features grouped by gene channel, shown across the identified clusters. Rows denote extracted features, and columns denote cells.

Conventionally, analysis of such imSRT data has relied on assigning and tallying the number of transcripts detected for each gene per segmented cell (Fig. 1A). This single-cell gene count matrix can then be used to identify transcriptionally distinct cell-types and states using dimensionality reduction and clustering approaches, analogous to conventional single-cell RNA-sequencing data analyses. However, these conventional analysis approaches inherently neglect the subcellular heterogeneity of these transcripts within cells.

Subcellular heterogeneity can play crucial roles in cellular function such that disruptions in subcellular RNA localization have been linked to diseases, including neurodegenerative diseases (Wu et al., 2020; Glock et al., 2017). For example, previous differential expression analysis of spatially-resolved RNA sequencing data has characterized the composition of synaptic RNAs in Alzheimer’s disease, implicating mislocalization of synaptic RNAs within neurons as a feature of a disease-associated cell-state (Smukowski et al., 2024). While imSRT offers transcriptome-wide profiling to map subcellular RNA heterogeneity, computational frameworks capable of systematically incorporating such information remain limited. Considering subcellular heterogeneity in the analysis of imSRT data may enable the identification of functionally distinct cellular states previously unrecognized through conventional gene count analysis.

## 2 Materials and Methods

As an alternative way to represent imSRT data to incorporate subcellular molecular spatial information, we developed STARIT (Spatial Transcriptomics As Rasterized Image Tensors) (Fig. 1B). STARIT creates image representations of intracellular spatial molecular distributions from imSRT data, using x and y coordinates of all molecules annotated with gene identity and cell assignment for each segmented cell. For each segmented cell, STARIT uses a previously developed rasterization strategy to generate one image per gene from the spatial coordinates of molecules (Clifton et al., 2023). Briefly, given the x-y coordinates of molecules within a cell, STARIT rasterizes molecular locations by encoding the presence of molecules within each pixel as bits, which are then convolved with a Gaussian blur kernel to produce a smooth image that captures local molecular density. The pixel resolution is defined such that one pixel corresponds to one unit in the input coordinate system. (Supplementary Methods). All rasterized gene images for each cell are combined, resulting in a tensor representation. These tensor representations can then be fed into deep learning computer vision models, such as the pre-trained ResNet101 model, for feature extraction, resulting in a single cell feature matrix representation that captures the spatial distribution and concentration of molecules within cells. Given this single-cell feature matrix, conventional single-cell analysis pipelines can then be applied to identify clusters of cells with similar features, potentially reflective of distinct subcellular heterogeneity.

## 3 Results

### 3.1 STARIT distinguishes cell states with subcellular molecular heterogeneity in simulated spatial transcriptomics data

To demonstrate the utility of STARIT for delineating cell states with subcellular molecular heterogeneity, beyond what conventional gene count clustering can capture, we first simulated imSRT data comprising of three transcriptionally distinct cell-types, each uniquely expressing one of three genes. For one cell-type, we further simulated cells such that they can be distinguished into two cell states based on nuclear versus perinuclear subcellular localization with no variation in expression magnitude between the two states. Specifically, we simulated cells of Group 1 to express Gene 1 uniquely, cells of Group 2 to express Gene 2 uniquely in a nuclear manner, cells of Group 3 to express Gene 2 uniquely in a perinuclear manner, and cells of Group 4 to express Gene 3 uniquely (Fig. 1C). When we represent this simulated imSRT data as conventional gene counts and perform PCA dimensionality reduction and Louvain graph-based clustering, we are only able to identify three clusters of cells that correspond to the three simulated transcriptionally distinct cell-types (Fig. 1D). This lack of correspondence between identified clusters and ground-truth groups can be further quantified via adjusted rand index (ARI) (score: 0.706) and the Jaccard similarity (mean: 0.833). While Cluster 1–1 corresponds perfectly to Group 1 cells and Cluster 1–0 to Group 4 cells, Cluster 1–2 contains a mixture of Group 2 and Group 3 cells. This overlap demonstrates that conventional representations of imSRT data and analyses are unable to distinguish between these two cell states, despite their distinct subcellular heterogeneity (Fig. 1E, Supplementary Fig. 1A). In contrast, when we apply STARIT, using the ResNet101 model for feature extraction followed by the same dimensionality reduction and clustering, we are able to identify four clusters of cells corresponding to the four simulated groups of cells representing cell-types and cell states with distinct subcellular heterogeneity (Fig. 1F, Supplementary Fig. 1B). This correspondence can be further quantified via ARI (score: 1.0) and in the on-diagonal Jaccard similarity (mean: 1.0) (Fig. 1G). We further perform the Wilcoxon-rank sum test on the ResNet101 features from STARIT between cells in the four identified clusters as an analogous analysis to conventional differential expression testing. We identified significant features associated with the expected genes differentiating the four identified clusters, suggesting that the extracted features are capturing aspects of both cellular and subcellular molecular heterogeneity (Fig. 1H). In this manner, STARIT enables an orthogonal approach for analyzing imSRT data to enable the identification and characterization of cell-states with distinct patterns of subcellular heterogeneity.

### 3.2 STARIT is sensitive to segmentation error, but can be remedied with gene-expression feature weighting

Having demonstrated the utility of STARIT on simulated data, we next sought to apply STARIT to real imSRT data. We applied STARIT to real imSRT data of the mouse cortex assayed by osmFISH, comprised of cell segmentation information and transcript spatial coordinates for 33 genes in 4,833 cells representing 31 cell-types (Supplementary Fig. 2) (Codeluppi et al., 2018). Applying STARIT with ResNet101, we derived a 4,833 cell by 67,584 feature matrix and followed with PCA dimensionality reduction, Louvain graph-based clustering, and UMAP visualization (Supplementary Fig. 3A). We identified 34 clusters using this approach and applied agglomerative clustering to obtain 31 clusters in an effort to recapitulate the original 31 cell-types identified by Codeluppi *et. al.* (Supplementary Fig. 3B). However, we observed poor correspondence between our clusters to the original 31 cell-types (Supplementary Fig. 3C). This lack of correspondence between identified clusters and ground-truth cell-types can be further quantified via ARI (score: 0.138) and in the on-diagonal Jaccard similarity (mean: 0.102). We hypothesized that this discrepancy is driven by minor segmentation errors, noisy gene detection, or other technical artifacts, whereby misassignment of a few molecules would substantially impact our extracted image features.

**Figure 2.**
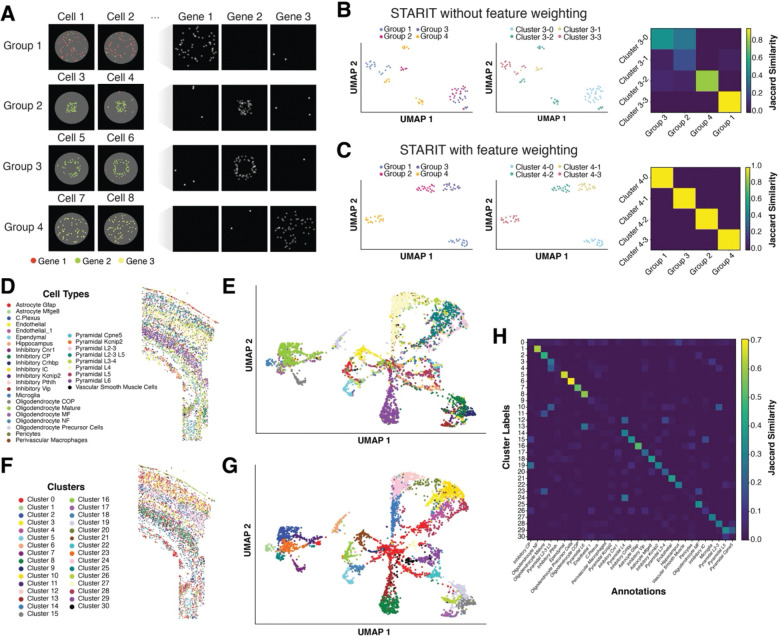
STARIT with feature weighting and application to osmFISH mouse cortex dataset. (A) Simulated imSRT data comprises four groups of cells that exhibit distinct subcellular molecular heterogeneity. Left: Example simulated cells from each group. All groups contain simulated noise across all genes. (B) STARIT analysis of simulated imSRT data with introduced noise, without feature weighting. Left: UMAP visualization of cells colored by ground-truth group annotation. Middle: UMAP visualization of cells colored by Louvain cluster assignment. Right: Jaccard similarity heatmap comparing ground-truth group annotations to Louvain clusters. (C) STARIT analysis of simulated imSRT data with introduced noise, with gene expression–based feature weighting. Left: UMAP visualization of cells colored by ground-truth group annotation. Middle: UMAP visualization of cells colored by Louvain cluster assignment. Right: Jaccard similarity heatmap comparing ground-truth group annotations to Louvain clusters. (D) Ground-truth cell-type annotations of osmFISH mouse somatosensory cortex dataset (Codeluppi et al., 2018) in tissue space. (E) UMAP visualization of osmFISH mouse somatosensory cortex ground-truth cell-types on ResNet101-extracted STARIT features. (F) STARIT-derived Louvain clustering of osmFISH mouse somatosensory cortex dataset in tissue space. (G) UMAP visualization of STARIT-derived Louvain clusters of osmFISH mouse somatosensory cortex from ResNet101-extracted STARIT features. (H) Jaccard similarity heatmap comparing ground-truth osmFISH mouse somatosensory cortex annotations to STARIT-derived Louvain clusters.

To illustrate this effect, we used a simulated imSRT dataset comprising once again of four groups of cells, now augmented with a few misassigned non-cell-type-specific molecules representing such minor segmentation errors and noisy gene detection (Supplementary Methods, Fig. 2A). When we apply STARIT to this simulated noisy imSRT data and perform PCA, Louvain graph-based clustering, and visualize in UMAP space, we can still identify four clusters of cells. However, these four clusters do not correspond to our four simulated groups of cells representing distinct cell-types and cell states (Fig. 2B). This lack of correspondence can be further quantified via ARI (score: 0.582) and in the on-diagonal Jaccard similarity (mean: 0.616). As such, this confirms how the presence of misallocated mRNA molecules, such as from minor segmentation errors or noisy gene detection, can have an impact on STARIT.

To address this issue, we introduced feature weighting to downweigh the contribution of such lowly detected genes in our STARIT analysis. Briefly, we multiply the extracted ResNet101 feature values for each gene in each cell by the min-max normalized expression magnitude of that gene in that cell (Supplementary Methods). Such feature weighting effectively incorporates the prior expectation that more highly expressed genes are more important in defining a cell-type or state, downweighing noisy or lowly expressed genes that may have been detected due to minor segmentation errors and noisy gene detection. Using feature weighting with our simulated noisy imSRT data, we are able to identify four clusters of cells corresponding perfectly to the four simulated groups of cells representing distinct cell-types and cell states as quantified via ARI (score: 1.0) and on-diagonal Jaccard similarity (mean: 1.0) (Fig. 2C). We further confirm that conventional gene counts clustering again identifies three clusters of cells, demonstrating that gene count clustering is more robust to such misassigned mRNA molecules but is still unable to resolve subcellular heterogeneity (Supplementary Fig. 4). This establishes that while STARIT is sensitive to segmentation error and gene detection noise, augmentation strategies such as weighting extracted features can be used to compensate to recapitulate expected results.

### 3.3 STARIT with feature weighting recapitulates expected cell-types

Having demonstrated that feature weighting is an effective strategy to handle potential minor segmentation errors and/or noisy gene detection, we incorporate such feature weighting into our analysis of the osmFISH data. With feature weighting, we observe improved correspondence between our identified clusters and the original cell-type annotations, as reflected in the similar patterns in tissue and UMAP space as well as improved ARI (score: 0.299) and higher on-diagonal Jaccard similarities (mean: 0.272) (Figs. 2D–H).

We do observe some new clusters unique to our STARIT analysis. For example, mature oligodendrocyte cells from the original osmFISH annotation are now split into clusters 2 and 23. As shown with our simulated data, such clusters may reflect distinct subcellular heterogeneity uniquely distinguishable through our STARIT analysis. We again used the Wilcoxon-rank sum test on the features between these two clusters to identify significantly differential features (Bonferroni-corrected p-value < 0.001) corresponding to the *Anln, Ctps, Gfap, Itpr2,* and *Plp1* genes’ STARIT representations.

To evaluate whether our newly identified clusters are solely due to STARIT or could be attributable to inherent instability in gene count clustering analysis, we performed PCA and UMAP dimensionality reduction followed by Louvain graph-based and agglomerative clustering to identify 31 clusters from re-clustering on the original gene counts matrix. We were able to observe similar clustering results for the gene counts re-clustering as from our STARIT analysis, including the splitting of mature oligodendrocyte cells (Supplementary Fig. 5). Consistent with this, when we used the Wilcoxon-rank sum test on the gene counts between the cells in the clusters 2 and 23 STARIT clusters, we were able to identify the same genes (*Anln, Ctps, Gfap, Itpr2, Plp1*) as significantly differentially expressed (Bonferroni-corrected p-value < 0.001). This suggests that while STARIT identified new cell clusters that split annotated mature oligodendrocytes and thus could correspond to distinct cell states, our re-clustering of gene counts demonstrates that such clustering differences could also be attributable to inherent instability in clustering. Nonetheless, this confirms that STARIT is able to capture comparable aspects of transcriptional heterogeneity as conventional gene count analysis and further confirms that image features extracted from STARIT can also capture magnitude differences in addition to subcellular heterogeneity to identify comparable differentially expressed genes driving cluster differences.

### 3.4 STARIT distinguishes bacterial states with rotational molecular heterogeneity in bacterial-MERFISH data

**Figure 3.**
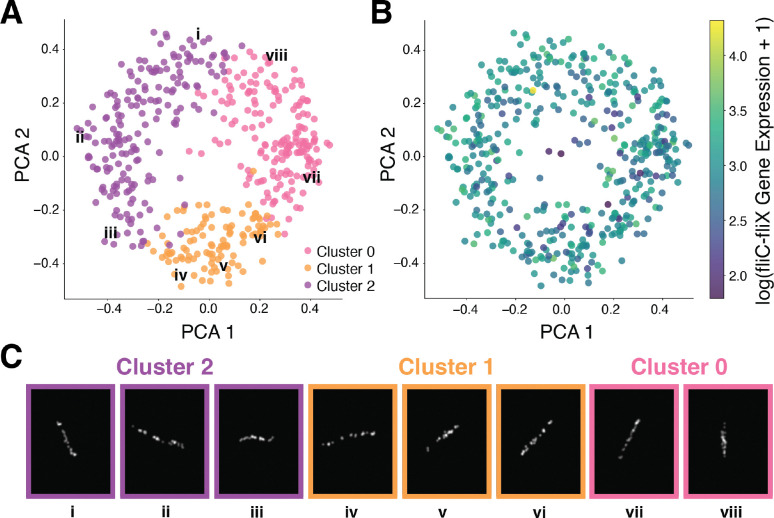
STARIT performance on bacterial-MERFISH 1000X data. (A) Principal component analysis (PCA) of STARIT-derived features from 463 *E. coli* cells based on fliC–fliX operon localization, imaged with bacterial-MERFISH at 1000X volumetric expansion. Cells are colored by Louvain graph-based cluster assignment: Cluster 0 (pink), Cluster 1 (orange), Cluster 2 (purple). Select labeled cells (i–viii) correspond to examples shown in panel C. (B) PCA embedding of cells colored by log-transformed fliC–fliX gene expression levels. (C) Representative rasterized fliC–fliX transcript distributions for cells i–viii labeled in panel A, with frame colors indicating cluster assignment.

We next sought to apply STARIT to real imSRT data of cultured cells representing one cell-type to identify possible subcellular heterogeneity suggestive of distinct cellular states. We applied STARIT to imSRT data of *E.coli* bacteria assayed by bacterial-MERFISH (Sarfatis et al., 2025). We focused on one 1000-fold volumetric expansion dataset, where the high expansion enabled reliable segmentation of bacteria confirmed by visual inspection. We performed quality control filtering to obtain 463 bacterial cells (Supplementary Methods). As a proof-of-concept, we focused on one assayed operon, fliC-fliX. We applied STARIT with ResNet101 to create a 463 cell by 2048 feature matrix, followed by PCA dimensionality reduction and Louvain graph-based clustering. We identified three clusters of bacteria using this approach that, when visualized in physical space, appear distinct in their spatial rotation of fliC-fliX molecules (Fig. 3A–C). Notably, these clusters of bacteria are not obviously distinguished based on fliC-fliX expression magnitude and therefore would not have been readily found via conventional count-based analysis (Fig. 3B). When mapped back to the culture dish, we can visually appreciate how bacteria from each cluster exhibit distinct spatial rotations (Supplementary Fig. 6). Therefore, in this example, the distinct spatial organization of fliC-fliX molecules is trivially driven by the rotation of the entire bacterium. Nonetheless, these results demonstrate that STARIT can resolve cells with distinct spatial heterogeneity from well-segmented imSRT data of cultured cells.

## 4 Discussion

Conventional representations of imSRT data, such as gene count matrices, inherently overlook spatial distributions of transcripts within cells, thereby inhibiting the identification of potential new cell states based on subcellular heterogeneity. We developed STARIT to represent imSRT data as image-based tensors that preserve such subcellular heterogeneity. On simulated imSRT data, STARIT combined with ResNet101 feature extraction and clustering analysis distinguished between cell states with distinct nuclear versus perinuclear molecular localization missed by conventional gene count clustering analysis. On real imSRT data, STARIT generally recapitulated expected cell-types in the mouse brain using osmFISH data and captured distinct heterogeneity in *E. coli* driven by rotation using bacterial-MERFISH data.

Despite these advances, several limitations remain. As we have demonstrated, STARIT relies heavily on accurate cell segmentation and detection specificity, as such segmentation errors and noisy transcripts can bias image-derived features. To mitigate these effects, STARIT currently employs feature weighting to downweigh the contribution of image features derived from lowly expressed genes. However, such feature weighting may mask true biology driven by subcellular localization of lowly expressed genes. Alternative feature weighting based on other quality metrics beyond expression magnitude may be used in the future. In general, these results are consistent with recognized persistent challenges in cell segmentation and detection specificity for imSRT data analysis (Yu et al., 2025; Hallinan et al., 2025). As imSRT technologies advance and segmentation quality improves for molecular resolution imSRT data, we expect the need for such feature weighting corrections to diminish.

Feature interpretability also presents an ongoing challenge. STARIT leverages deep learning-based feature extraction, which functions as a “black box” with limited biological interpretability. While differential testing can identify features that significantly differ between clusters, as we have demonstrated, their biological meaning often remains unclear. Likewise, while explainable AI approaches such as GradCAM and GAINext can provide coarse localization of model attention, they do not explain why specific features drive downstream cluster assignments (Selvaraju et al., 2017; Li et al., 2018). We anticipate that additional supervised and semi-supervised statistics regarding subcellular molecular organization could also be incorporated to further interpret cluster differences (Bierman et al., 2024; Mah et al., 2024; Wang and Zhou, 2025).

While we have focused on demonstrating applications of STARIT in conjunction with a pre-trained ResNet-based feature extraction, other deep learning-based feature extraction can also be used. Because ResNet lacks built-in rotational invariance, we distinguished bacteria with varying rotations in our bacterial-MERFISH data analysis. In general, such sensitivity of ResNet extracted features to rotation may limit analyses of polarized structures such as apical–basal organization in epithelia. As such, combining STARIT with rotationally invariant or biological domain-specific models such as SubCell, UNI, and CTransPath for feature extraction as well as targeted data augmentations, may be explored in the future to improve robustness (Wang et al., 2022; Gupta et al., 2024; Chen et al., 2024).

Looking forward, by providing an alternative image-based representation of imSRT data, STARIT opens opportunities to integrate image-based representations of imSRT data with many downstream applications. Emerging frameworks such as scPortrait, a scverse-native toolkit that standardizes single-cell microscopy images in an AnnData-compatible format, illustrate how STARIT and related rasterization workflows could be wrapped into interoperable packages within broader computational infrastructure (Mädler et al., 2025). More broadly, as imSRT technologies advance and cell segmentation improves, and likewise computer vision models advance and interpretable feature extraction improves, we anticipate that image-based representations of spatial transcriptomics will become a useful approach for resolving subcellular organization and for generating biologically meaningful interpretations. Overall, we anticipate that STARIT will enhance the identification and characterization of novel cell-types and states by capturing subcellular molecular heterogeneity, providing deeper insights into cellular organization and functional diversity that were overlooked by gene count analysis methods.

## Supplementary Material

Supplement 1

## Data Availability

All data analyzed with STARIT are publicly available. The simulated datasets are available at https://github.com/JEFworks-Lab/STARIT/tree/main/data. The osmFISH datasets are available at https://linnarssonlab.org/osmFISH/availability/. The Bacterial-MERFISH dataset is available at https://datadryad.org/dataset/doi:10.5061/dryad.n5tb2rc4d.

## References

[R1] Bierman R. (2024) Statistical analysis supports pervasive RNA subcellular localization and alternative 3’ UTR regulation. eLife, 12, RP87517. 39699003 10.7554/eLife.87517 PMC11658768

[R2] Chen R.J. (2024) Towards a general-purpose foundation model for computational pathology. Nat. Med., 30, 850–862. 38504018 10.1038/s41591-024-02857-3 PMC11403354

[R3] Clifton K. (2023) STalign: Alignment of spatial transcriptomics data using diffeomorphic metric mapping. Nat. Commun., 14, 8123. 38065970 10.1038/s41467-023-43915-7 PMC10709594

[R4] Codeluppi S. (2018) Spatial organization of the somatosensory cortex revealed by osmFISH. Nat. Methods, 15, 932–935. 30377364 10.1038/s41592-018-0175-z

[R5] Glock C. (2017) mRNA transport & local translation in neurons. Curr. Opin. Neurobiol., 45, 169–177. 28633045 10.1016/j.conb.2017.05.005

[R6] Gupta A. (2024) SubCell: Vision foundation models for microscopy capture single-cell biology. 2024.12.06.627299.

[R7] Hallinan C. (2025) Evidence of off-target probe binding in the 10x Genomics Xenium v1 Human Breast Gene Expression Panel compromises accuracy of spatial transcriptomic profiling. eLife, 14. 10.7554/eLife.107070 PMC13134852 42065925

[R8] Li K. (2018) Tell Me Where to Look: Guided Attention Inference Network.

[R9] Mädler S.C. (2025) scPortrait integrates single-cell images into multimodal modeling. 2025.09.22.677590.

[R10] Mah C.K. (2024) Bento: a toolkit for subcellular analysis of spatial transcriptomics data. Genome Biol., 25, 82. 38566187 10.1186/s13059-024-03217-7 PMC11289963

[R11] Petukhov V. (2022) Cell segmentation in imaging-based spatial transcriptomics. Nat. Biotechnol., 40, 345–354. 34650268 10.1038/s41587-021-01044-w

[R12] Sarfatis A. (2025) Highly multiplexed spatial transcriptomics in bacteria. Science, 387, eadr0932. 39847624 10.1126/science.adr0932 PMC12278067

[R13] Selvaraju R.R. (2017) Grad-CAM: Visual Explanations from Deep Networks via Gradient-Based Localization. In, 2017 IEEE International Conference on Computer Vision (ICCV)., pp. 618–626.

[R14] Smukowski S.N. (2024) mRNA and circRNA mislocalization to synapses are key features of Alzheimer’s disease. PLOS Genet., 20, e1011359. 39074152 10.1371/journal.pgen.1011359 PMC11309398

[R15] Stringer C. (2021) Cellpose: a generalist algorithm for cellular segmentation. Nat. Methods, 18, 100–106. 33318659 10.1038/s41592-020-01018-x

[R16] Wang J.X. and Zhou X. (2025) ELLA: modeling subcellular spatial variation of gene expression within cells in high-resolution spatial transcriptomics. Nat. Commun., 16, 9920. 41219206 10.1038/s41467-025-64867-0 PMC12606177

[R17] Wang X. (2022) Transformer-based unsupervised contrastive learning for histopathological image classification. Med. Image Anal., 81, 102559. 35952419 10.1016/j.media.2022.102559

[R18] Wu K.E. (2020) RNA-GPS predicts high-resolution RNA subcellular localization and highlights the role of splicing. RNA, 26, 851–865. 32220894 10.1261/rna.074161.119 PMC7297119

[R19] Yu H. (2025) Segmentation Matters: Recognizing the Cell Segmentation Challenge in Spatial Transcriptomics. 2025.08.25.672145.

[R20] Zhuang X. (2021) Spatially resolved single-cell genomics and transcriptomics by imaging. Nat. Methods, 18, 18–22. 33408406 10.1038/s41592-020-01037-8 PMC9805800

