# Supplementary material for: Spatial Transcriptomics As Rasterized Image Tensors (STARIT) characterizes cell states with subcellular molecular heterogeneity
Source: bioRxiv. 2025 Dec 22:2025.12.18.695193. Preprint. [Version 1] doi: 10.64898/2025.12.18.695193 (PMC12767309; doi:10.64898/2025.12.18.695193)
Supplement: Supplement 1 [file NIHPP2025.12.18.695193v1-supplement-1.pdf]

bioRxiv preprint doi: https://doi.org/10.64898/2025.12.18.695193; this version posted December 22, 2025. The copyright holder for this preprint (which was not certified by peer review) is the author/funder, who has granted bioRxiv a license to display the preprint in perpetuity. It is made available under aCC-BY 4.0 International license.

Supplementary Methods

1. Spatial Transcriptomics As Rasterized Image Tensors (STARIT)

Given three inputs, (i) *bounding_box,* (ii) x, and (iii) y, STARIT can transform these coordinates into a density image. The first input *bounding_box* allows for maintenance of a cell-aspect ratio and is generated from a helper function. The axis-aligned bounding box of the coordinates is computed by finding the min and max of x and y. The box bounded by these four values is then expanded by a factor called expand (default 1.1) to provide a small margin for the rasterized image. The second and third inputs are the (x,y) molecular coordinates you wish to rasterize for a given cell. The bounding box image grid is then defined at a desired pixel resolution size (default 1.0, in the same units as the input coordinates). From there, each gene molecule is then rendered to the canvas grid by convolving a normalized Gaussian kernel, blur (default 1.0), centered at (x,y). The pixelated image is created by summing contributions from all gene molecules in the cell. The result is STARIT producing one PNG image per gene per cell. When a gene has no molecules, a blank image of the canonical size is saved to preserve the tensor shape.

2. Simulated imSRT Data

Simulation Framework with Manually Defined Groups and Sub-cellular Spatial Patterns

To evaluate STARIT under controlled conditions, we generated a fully synthetic imSRT dataset with a known ground-truth spatial organization. We simulated four groups, each containing 20 circular “cells” represented within a 224x224 pixel image. For each cell, we simulated the spatial locations of transcripts for three genes, with each gene exhibiting a distinct, group-specific spatial pattern. Specifically, we generated 50 transcript coordinates for gene-group pairs defined to be expressed in that group. In Group 1 (gene 1) and Group 4 (gene 3), transcripts were uniformly sampled from anywhere within the full circular cell (radius = 75 pixels), resulting in a random cell-wide spatial pattern. In Group 2 (gene2), transcripts

bioRxiv preprint doi: https://doi.org/10.64898/2025.12.18.695193; this version posted December 22, 2025. The copyright holder for this preprint (which was not certified by peer review) is the author/funder, who has granted bioRxiv a license to display the preprint in perpetuity. It is made available under aCC-BY 4.0 International license.

were restricted to a smaller central region (radius = 25 pixels), producing a spatial pattern that reflects the nuclear space. In Group 3 (gene 2), transcripts were sampled from an annular region between circle radii of 25 and 45 pixels, yielding a perinuclear ring-like spatial pattern. The final simulated dataset consisted of transcript-level x, y coordinates annotated with gene, cell, and group identity, along with the derived traditional cell-by-gene count matrix for downstream evaluation and comparison.

To further evaluate the robustness of STARIT to low-level background signal, we generated a second version of the synthetic dataset that introduced sparse noise transcripts. Noise was added only to gene-group combinations in a non-cell-type-specific manner (e.g., Gene 2 transcripts added to Group 1 cells). For these non-expressing gene-group pairs, we added zero to three randomly sampled transcript coordinates per cell, drawn uniformly from anywhere within the cell boundary (radius = 75 pixels). For example, in Groups 2 and 3, where only gene 2 is truly expressed, we added a small number of noise transcripts for gene 1 and gene 3. Similarly, in Group 1, we added noise for gene 2 and gene 3, and in Group 4, we added noise for gene 1 and gene 2. All spatial patterns that defined each group remained unchanged between the two synthetic datasets, as did the final simulated output structure.

Applying STARIT to Simulated imSRT Data

STARIT was applied to each simulated dataset to generate one image per cell-gene pair, with a pixel resolution size (dx) of 1 and a Gaussian kernel (blur) of 1. Because each cell contains three genes, three corresponding images were produced; if a cell had zero expression for a given gene, a black image was used in its place.

Downstream Analysis of the Simulated imSRT Datasets

bioRxiv preprint doi: https://doi.org/10.64898/2025.12.18.695193; this version posted December 22, 2025. The copyright holder for this preprint (which was not certified by peer review) is the author/funder, who has granted bioRxiv a license to display the preprint in perpetuity. It is made available under aCC-BY 4.0 International license.

All downstream analysis steps and parameters described below were applied identically to both simulated datasets with and without noise.

Each STARIT image was converted to RGB, resized to 224x224 pixels with aspect-ratio preservation and padding, normalized with ImageNet preprocessing (mean= [0.485, 0.456, 0.406], std= [0.229, 0.224, 0.225]), and processed using a pretrained ResNet101 for feature extraction. The 2048-dimensional ResNet features extracted from each gene-specific image were concatenated to form a 6144-dimensional feature vector per cell (3 genes x 2,048 features). Feature vectors from all 80 cells were then stacked into an 80 x 6144 matrix, which served as the input for all downstream analyses.

We first performed Principal Components Analysis (PCA) and retained the top 5 principal components (PCs). These PCs were then used as input to Uniform Manifold Approximation and Projection (UMAP), using nearest neighbors of 30 and a minimum distance of 0.1, keeping all other settings at their defaults. For clustering, we constructed a k-nearest neighbors (kNN) graph on the PCs using cosine distance, 18 nearest neighbors, including self to true, and connectivity mode, keeping all other settings at their defaults, and applied Louvain graph-based clustering with default parameters. To quantify agreement between unsupervised clusters and ground-truth groups, we computed a Jaccard similarity matrix for all cells. In addition to pairwise Jaccard scores, we assessed global clustering accuracy using the adjusted rand index (ARI) to quantify the similarity between two complete partitions while correcting for chance agreement in order to summarize the overall correspondence between inferred clusters and the curated ground-truth annotations.

To visualize the full set of learned image features, we generated a heatmap representing all 6,144 features (3 genes x 2,048 features) across all cells. Feature values were standardized across cells using z-score normalization, followed by hierarchical clustering with Ward’s linkage and Euclidean distance.

Gene Count Analysis of Simulated imSRT Data

bioRxiv preprint doi: https://doi.org/10.64898/2025.12.18.695193; this version posted December 22, 2025. The copyright holder for this preprint (which was not certified by peer review) is the author/funder, who has granted bioRxiv a license to display the preprint in perpetuity. It is made available under aCC-BY 4.0 International license.

For the gene-count analysis, PCA was performed on the normalized gene-count matrix instead of the cell-by-feature matrix and retained the top 3 PCs. These PCs were then used as input to UMAP using nearest neighbors of 20 and a minimum distance of 0.5, keeping all other settings at their defaults. For clustering, we constructed a kNN graph on the PCs using cosine distance, 20 nearest neighbors, including self to true, and connectivity mode, keeping all other settings at their defaults, and applied Louvain graph-based clustering with default parameters.

For the feature-weighting analysis on the simulated data with noise, we first performed PCA and retained the top 5 PCs. These PCs were then used as input to UMAP using nearest neighbors of 10 and a minimum distance of 0.5, keeping all other settings at their defaults. For clustering, we constructed a kNN graph on the PCs using cosine distance, 8 nearest neighbors, including self to true, and connectivity mode, keeping all other settings at their defaults, and applied Louvain graph-based clustering with default parameters. To quantify agreement between unsupervised clusters and ground-truth groups, we computed a Jaccard similarity matrix for all cells.

3. osmFISH Mouse Cortex Data

Data Acquisition for osmFISH Data

The osmFISH mouse cortex dataset was obtained from the original publication Codeluppi, *et al.* (Codeluppi *et al.*, 2018).

Preprocessing osmFISH Data

We removed cells with associated regions designated as “Excluded” in the original annotation, as well as 6 additional cells with none of the 33 assayed genes detected. This resulted in 4,833 cells with 33 genes across 31 cell types. Subcellular coordinates were centered at each cell’s origin.

Applying STARIT to osmFISH Data

bioRxiv preprint doi: https://doi.org/10.64898/2025.12.18.695193; this version posted December 22, 2025. The copyright holder for this preprint (which was not certified by peer review) is the author/funder, who has granted bioRxiv a license to display the preprint in perpetuity. It is made available under aCC-BY 4.0 International license.

STARIT was applied to all 4,833 cells and all 33 genes using a pixel resolution size (dx) of 1 and a Gaussian kernel (blur) of 1. A total of 159,489 gene-specific images were generated (4,833 cells x 33 genes). For genes with zero detected molecules in a given cell, a black 224x224 pixel image was produced.

Downstream osmFISH Analysis with No Feature Weighting

Each STARIT image was converted to RGB, resized to 224x224 pixels with aspect-ratio preservation and padding, and normalized with ImageNet. A pretrained ResNet101 with the final fully connected layer removed was used to extract a 2048-dimensional feature embedding for each gene image. Because there are thirty-three gene markers, 33 corresponding images were produced. The 2048-dimensional ResNet features extracted from each gene-specific image were concatenated to form a 67,584-dimensional feature vector per cell (33 genes x 2048 features). Feature vectors from all 4,833 cells were then stacked into a 4,833 x 67,584 matrix, which served as the input for all downstream analyses.

We performed PCA and retained the top 30 PCs. These PCs were then used as input to UMAP using a cosine metric, nearest neighbors of 50, and a minimum distance of 0.15. For clustering, we constructed a kNN graph on the PCs using cosine distance and 5 nearest neighbors, include self to true, and connectivity mode, keeping all other settings at their defaults, and applied Louvain graph-based clustering with default parameters. To match the 31 curated cell types, we performed hierarchical agglomerative clustering on Louvain community centroids in PCA space to obtain 31 meta-clusters. Agreement with ground-truth cell type labels was quantified using a Jaccard similarity matrix and ARI.

Downstream osmFISH Analysis with Feature Weighting

Using the original osmFISH cell-by-gene count matrix, gene counts were min–max scaled per gene across cells. As in the non-weighted analysis, a 2048-dimensional feature embedding was extracted for each

bioRxiv preprint doi: https://doi.org/10.64898/2025.12.18.695193; this version posted December 22, 2025. The copyright holder for this preprint (which was not certified by peer review) is the author/funder, who has granted bioRxiv a license to display the preprint in perpetuity. It is made available under aCC-BY 4.0 International license.

gene image, for all 33 genes in all 4,833 cells. We applied per-gene feature weighting on the gene feature embeddings. For each cell and gene, the corresponding 2048-dimensional image embedding was multiplied by the scaled gene count value to generate feature-weighted embeddings. Weighted embeddings were concatenated in the same manner as the unweighted embeddings to yield a feature-weighted cell-by-feature matrix of dimension 4,833 x 67,584.

PCA and UMAP were applied to the weighted features using the same parameters as with the non-weighted downstream analysis. Louvain graph-based clustering was performed on a kNN graph constructed with 6 neighbors, include self to true, and connectivity mode, followed by hierarchical clustering to obtain 31 meta-clusters. Cluster–ground-truth label agreement was again quantified using Jaccard similarity and ARI.

Gene Counts Analysis for osmFISH Data

Using the original osmFISH cell-by-gene count matrix, gene counts were filtered identically and min–max scaled per gene across cells (4,833 cells x 33 genes). We first performed PCA and retained the top 30 PCs. These PCs were then used as input to UMAP using a cosine metric, nearest neighbors of 50, and a minimum distance of 0.15. For clustering, we constructed a kNN graph on the PCs using 6 nearest neighbors, include self to true, and connectivity mode, keeping all other settings at their defaults, and applied Louvain graph-based clustering with a resolution of 1.2 and kept all other default parameters. To match the 31 curated cell types, we performed hierarchical agglomerative clustering on Louvain community centroids in PCA space to obtain 31 meta-clusters. Agreement with ground-truth cell type labels was quantified using a Jaccard similarity matrix and an ARI.

5. Bacteria-MERFISH Data

bioRxiv preprint doi: https://doi.org/10.64898/2025.12.18.695193; this version posted December 22, 2025. The copyright holder for this preprint (which was not certified by peer review) is the author/funder, who has granted bioRxiv a license to display the preprint in perpetuity. It is made available under aCC-BY 4.0 International license.

Data Acquisition of Bacterial-MERFISH Data

The 1000-fold volumetric expansion bacteria-MERFISH dataset was obtained from the original publication Sarfatis *et al.* at https://datadryad.org/dataset/doi:10.5061/dryad.n5tb2rc4d (Sarfatis *et al.*, 2025).

Preprocessing Bacterial-MERFISH Data

For the 1000-fold bacterial-MERFISH dataset, we filtered for bacterial cells with more than 250 total mRNA molecules detected, narrowing down to 463 cells. For each cell, molecular coordinates were subsetted to those corresponding to the fliC–fliX operon. Subcellular coordinates were centered at each cell's origin.

Applying STARIT to Bacterial-MERFISH Data

STARIT was applied to these centered fliC-fliX molecular coordinates, with a pixel resolution size (dx) of 1 and a Gaussian kernel (blur) of 1, producing one STARIT image per cell (n = 463). A total of 463 gene image representations of fliC-fliX were created.

Downstream Analysis of Bacterial-MERFISH Data

Each STARIT image was converted to RGB, resized to 224×224 pixels with aspect-ratio preservation and padding, and normalized with ImageNet preprocessing. A pretrained ResNet101 with the final fully connected layer removed was used to extract a 2048-dimensional feature embedding for each operon image to form a 2048-dimensional feature vector per cell (1 operon x 2,048 features). Feature vectors from all 463 cells were then stacked into a 463 x 2,048 matrix, which served as the input for all downstream analyses.

bioRxiv preprint doi: https://doi.org/10.64898/2025.12.18.695193; this version posted December 22, 2025. The copyright holder for this preprint (which was not certified by peer review) is the author/funder, who has granted bioRxiv a license to display the preprint in perpetuity. It is made available under aCC-BY 4.0 International license.

We performed PCA and retained the top 30 PCs. For clustering, we constructed a kNN graph on the PCs using cosine distance, connectivity mode, include self to true, and 100 nearest neighbors, keeping all other settings at their defaults, and applied Louvain graph-based clustering with default parameters.

Cluster-specific variation in fliC–fliX expression was visualized using PCA embeddings colored by raw and log-transformed counts.

bioRxiv preprint doi: https://doi.org/10.64898/2025.12.18.695193; this version posted December 22, 2025. The copyright holder for this preprint (which was not certified by peer review) is the author/funder, who has granted bioRxiv a license to display the preprint in perpetuity. It is made available under aCC-BY 4.0 International license.

Supplementary Figures

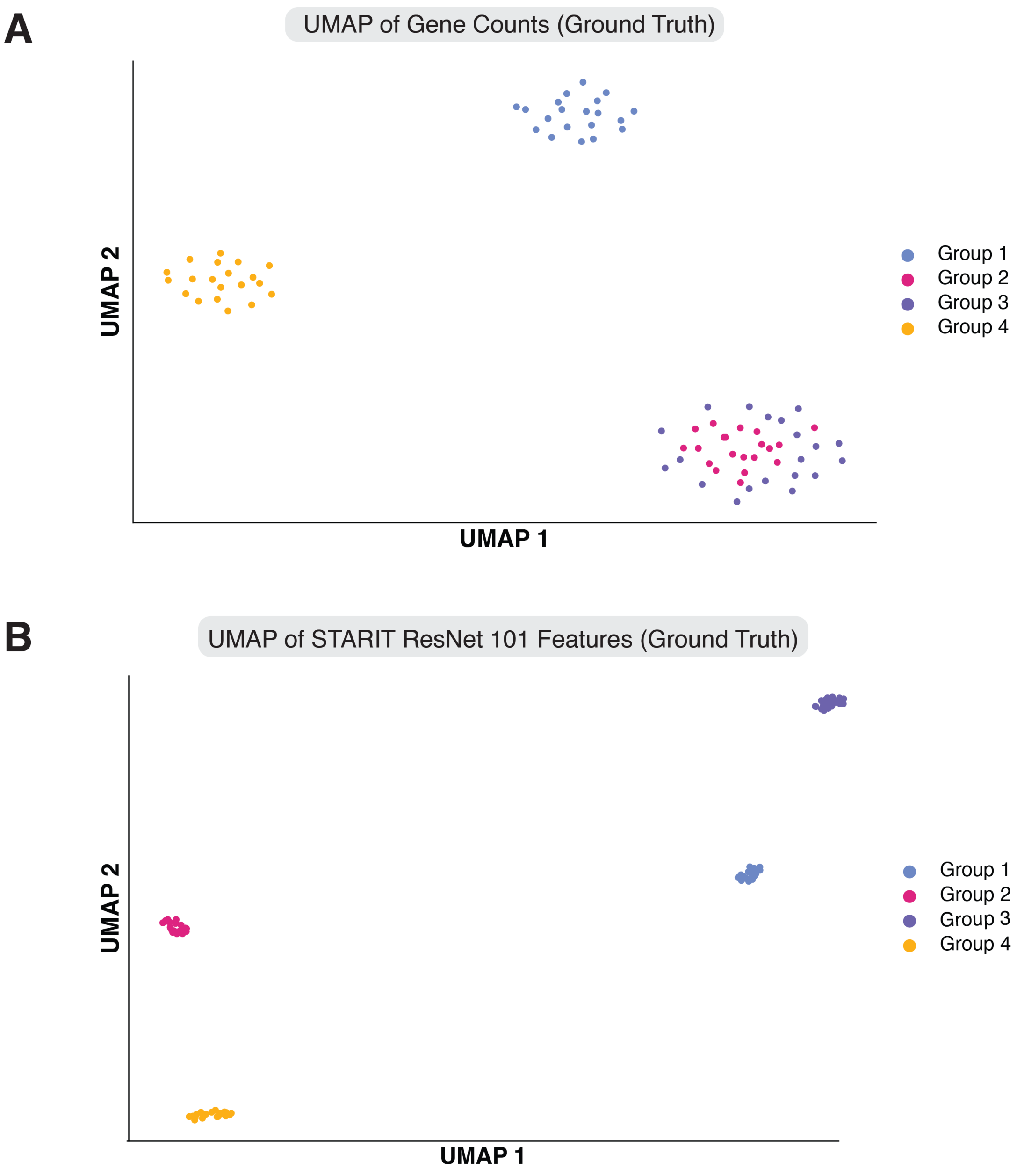

Supplementary Figure 1. Comparison of ground-truth labels on UMAPs derived from gene counts versus STARIT-derived ResNet101 features for simulated imSRT data with subcellular heterogeneity. (A) UMAP embedding of PCs from the gene count matrix with ground-truth annotations of simulated groups (B) UMAP embedding of PCs from STARIT-derived ResNet101 image features with ground-truth annotations of simulated groups.

bioRxiv preprint doi: https://doi.org/10.64898/2025.12.18.695193; this version posted December 22, 2025. The copyright holder for this preprint (which was not certified by peer review) is the author/funder, who has granted bioRxiv a license to display the preprint in perpetuity. It is made available under aCC-BY 4.0 International license.

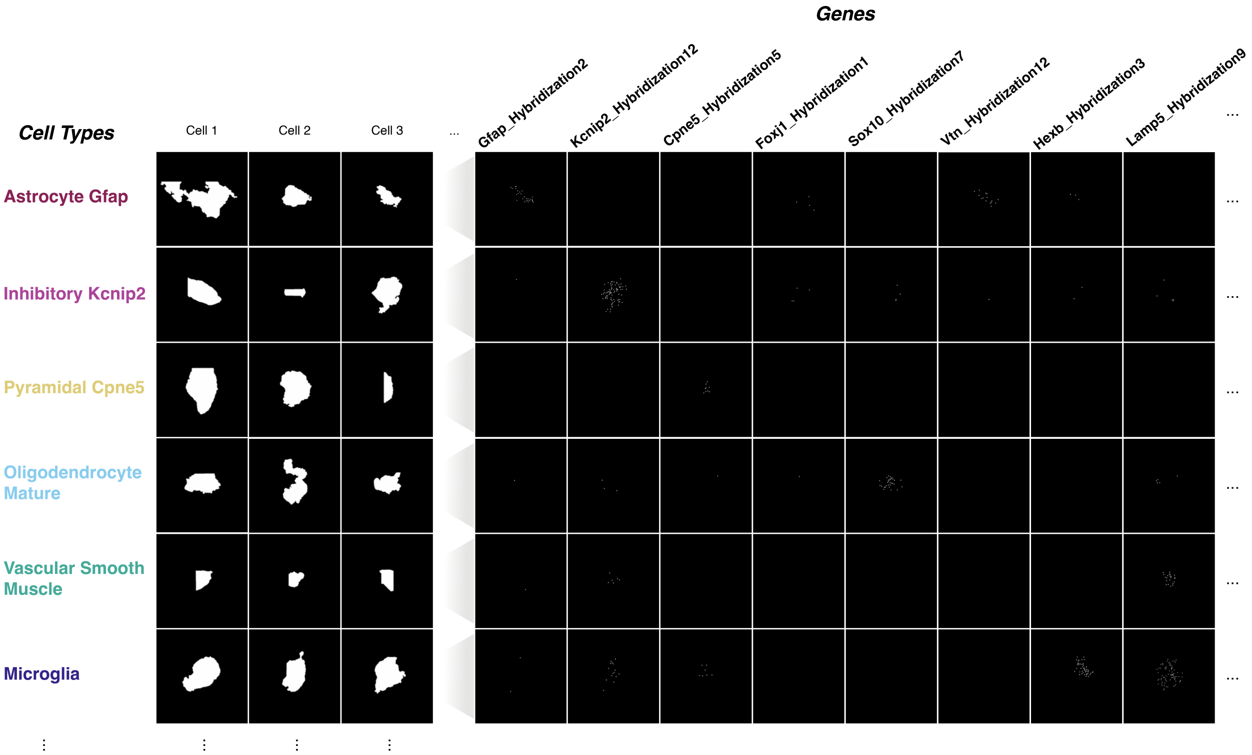

Supplementary Figure 2. Example cell segmentation and gene-specific STARIT rasterized images from osmFISH mouse cortex data cell types. Representative cell segmentation and corresponding STARIT-generated gene rasterizations for selected osmFISH-defined cell types. Left: For each of six example cell types (Astrocyte Gfap, Inhibitory Kcnip2, Pyramidal Cpne5, Oligodendrocyte Mature, Vascular Smooth Muscle, and Microglia), we show three individual cell segmentation masks. Right: For one of the cells of each cell type, we display STARIT gene tensor rasterizations for a subset of the 33 assayed genes, where each panel shows the spatial distribution of transcripts within the cell segmentation mask boundary.

bioRxiv preprint doi: https://doi.org/10.64898/2025.12.18.695193; this version posted December 22, 2025. The copyright holder for this preprint (which was not certified by peer review) is the author/funder, who has granted bioRxiv a license to display the preprint in perpetuity. It is made available under aCC-BY 4.0 International license.

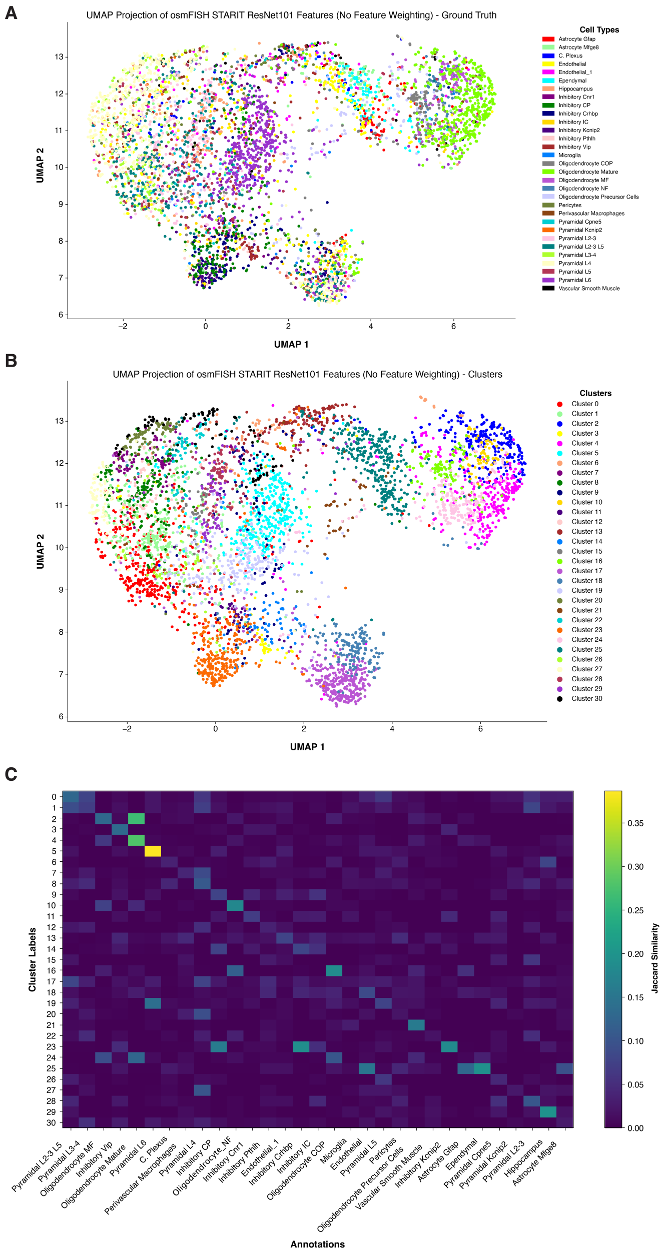

Supplementary Figure 3. STARIT feature embedding and clustering of osmFISH mouse cortex cells and comparison to previously annotated cell types, without feature weighting. (A) UMAP visualization of STARIT-derived features for 4,833 osmFISH cells from the mouse cortex, colored by the 31 original cell-type annotations reported by Codeluppi *et al.* (2018). Each point represents a single cell. (B) UMAP embedding of the same STARIT features colored by 31 STARIT-derived Louvain clusters and subsequent agglomerative clustering. Each point represents a single cell. (C) Jaccard similarity matrix comparing STARIT-derived Louvain clusters (rows) to ground-truth cell-type annotations (columns).

bioRxiv preprint doi: https://doi.org/10.64898/2025.12.18.695193; this version posted December 22, 2025. The copyright holder for this preprint (which was not certified by peer review) is the author/funder, who has granted bioRxiv a license to display the preprint in perpetuity. It is made available under aCC-BY 4.0 International license.

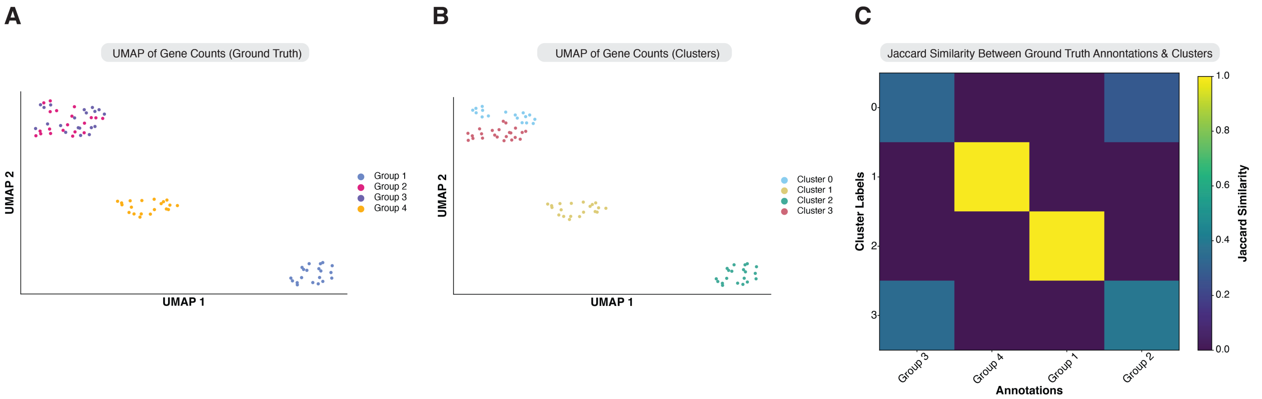

Supplementary Figure 4. Effect of misallocated mRNA molecules on conventional gene-count–based clustering on simulated noisy imSRT data. (A) UMAP embedding of PCs from the gene count matrix is shown for ground-truth simulated noisy imSRT group annotations. (B) UMAP embedding of PCs from the gene count matrix is shown for Louvain clusters. (C) Jaccard similarity heatmap compares Louvain clusters and ground-truth simulated noisy imSRT group annotations.

bioRxiv preprint doi: https://doi.org/10.64898/2025.12.18.695193; this version posted December 22, 2025. The copyright holder for this preprint (which was not certified by peer review) is the author/funder, who has granted bioRxiv a license to display the preprint in perpetuity. It is made available under aCC-BY 4.0 International license.

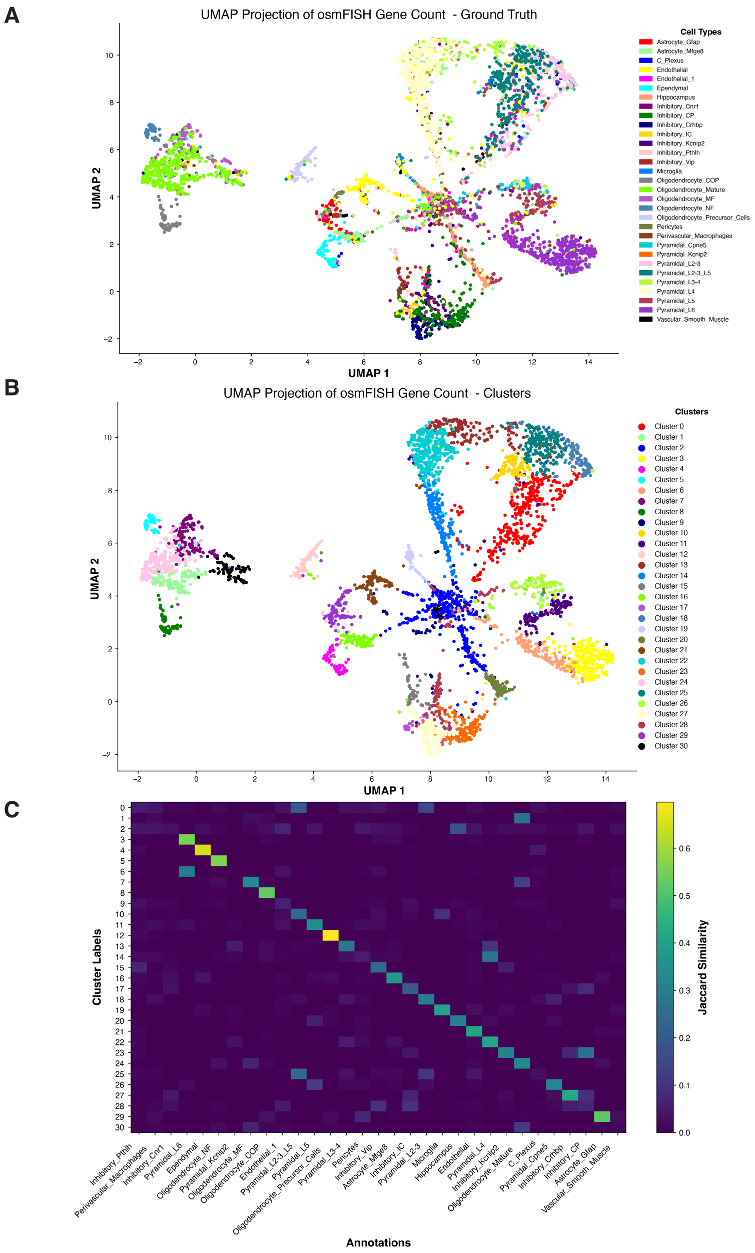

Supplementary Figure 5. Gene count–based clustering of osmFISH mouse cortex data. (A) UMAP embedding of PCs from the gene count matrix of the osmFISH mouse cortex dataset, colored by the 31 previously annotated cell types from *Codeluppi et al.* (2018). (B) UMAP embedding of the same gene count–derived features colored by 31 Louvain clusters from STARIT-derived ResNet101 image features followed by agglomerative clustering. (C) Jaccard similarity matrix comparing gene count–derived Louvain clusters (rows) to annotated cell types (columns).

bioRxiv preprint doi: https://doi.org/10.64898/2025.12.18.695193; this version posted December 22, 2025. The copyright holder for this preprint (which was not certified by peer review) is the author/funder, who has granted bioRxiv a license to display the preprint in perpetuity. It is made available under aCC-BY 4.0 International license.

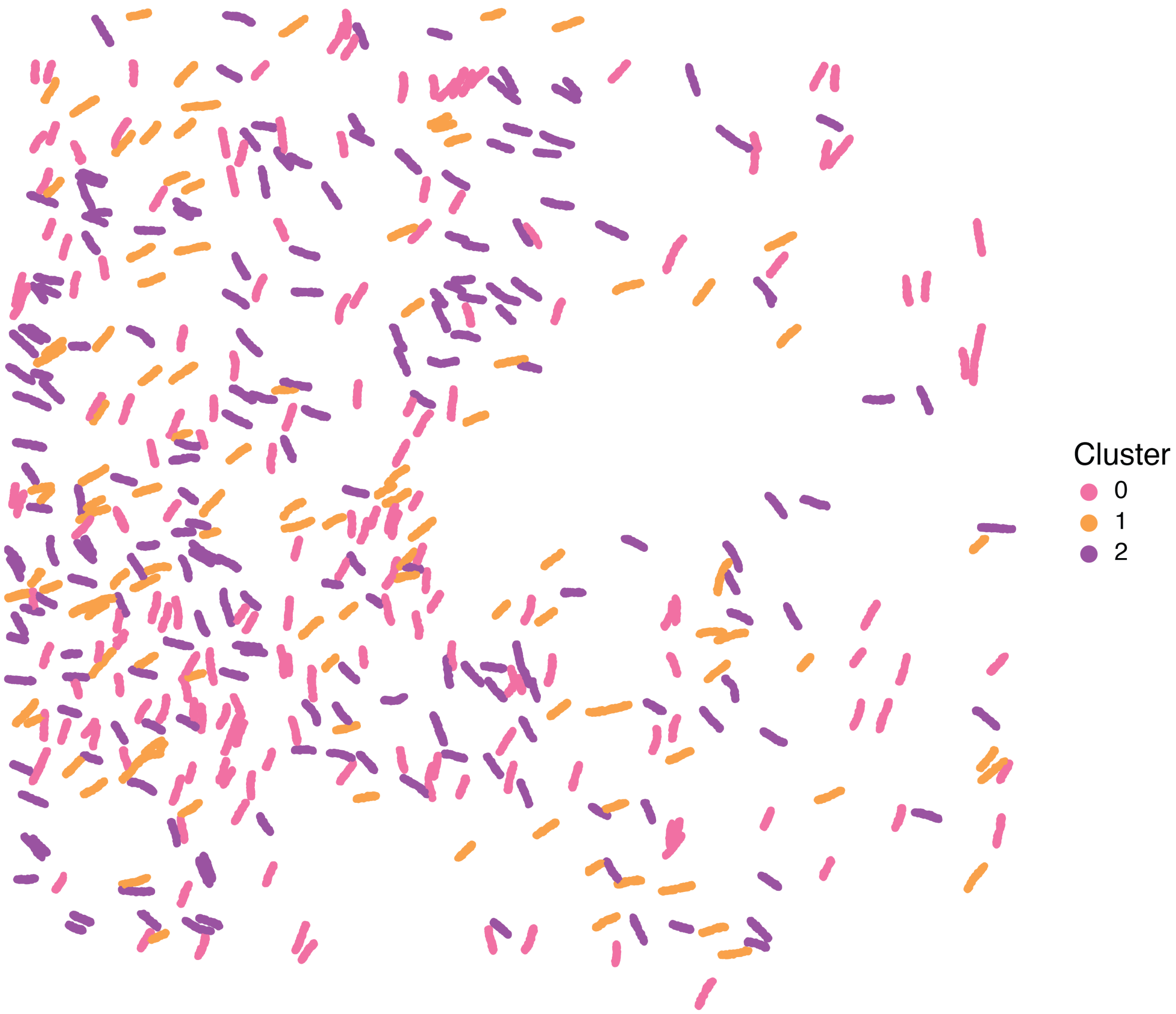

Supplementary Figure 6. Physical-space visualization of STARIT-identified clusters in Bacterial-MERFISH *E. coli* data. Shown are the spatial positions of 463 *E. coli* cells assayed by Bacterial-MERFISH after 1000-fold volumetric expansion, plotted in their original physical coordinates and colored by the three Louvain clusters identified from STARIT-derived ResNet101 image features.
